# Retraction: Ag@TiO_2_ nanocomposite; synthesis, characterization and its application as a novel and recyclable catalyst for the one-pot synthesis of benzoxazole derivatives in aqueous media

**DOI:** 10.1039/d5ra90052b

**Published:** 2025-05-02

**Authors:** Behrooz Maleki, Mehdi Baghayeri, Seyed Mohammad Vahdat, Abbas Mohammadzadeh, Somaieh Akhoondi

**Affiliations:** a Department of Chemistry, Hakim Sabzevari University Sabzevar 96179-76487 Iran b.maleki@hsu.ac.ir +98 5144013324; b Department of Chemistry, Islamic Azad University, Ayatollah Amoli Branch P.O. Box 678 Amol Iran; c Department of Physics, Nour Branch, Islamic Azad University Nour Iran

## Abstract

Retraction of ‘Ag@TiO_2_ nanocomposite; synthesis, characterization and its application as a novel and recyclable catalyst for the one-pot synthesis of benzoxazole derivatives in aqueous media’ by Behrooz Maleki *et al.*, *RSC Adv.*, 2015, **5**, 46545–46551, https://doi.org/10.1039/C5RA06618B.

The Royal Society of Chemistry hereby wholly retracts this *RSC Advances* article due to concerns with the reliability of the data.

There are concerns with the reliability of the XRD data in Fig. 4, and the raw data supplied by the authors. The authors initially provided raw data for trace a, but this did not match the published data. When questioned, the authors re-supplied what they claimed to be the same data alongside traces b, c, and d. However further discrepancies were found between the data sets, and no data provided matched the published data. The discrepancies in the data have not been explained, and these changes indicate signs of data manipulation or generation.

Given the significance of these concerns, the Editor has lost confidence that the findings presented in this paper are reliable.

The authors were informed about the retraction of the article. Behrooz Maleki has not agreed with the decision, the other authors have not responded.

Signed: Laura Fisher, Executive Editor, *RSC Advances*

Date: 25th April 2025

